# Does alternation of *Candida albicans TUP1* gene expression affect the progress of symptomatic recurrent vulvovaginal candidiasis?

**DOI:** 10.18502/CMM.6.2.2694

**Published:** 2020-06

**Authors:** Mona Ghazanfari, Azam Fattahi, Mehraban Falahati, Majid Bakhshizadeh, Maryam Roudbary, Faramarz Masjedian Jazi, Mohsen Keykhosravi, Ensieh Lotfali

**Affiliations:** 1 Department of Medical Mycology, Faculty of Medical Sciences, Iran University of Medical Sciences, Tehran, Iran; 2 Invasive Fungi Research Centre,Communicable Diseases Institute Department of Medical Mycology, School of Medicine, Mazandaran University of Medical Sciences, Sari, Iran; 3 Student Research Committee , Mazandaran University of Medical Sciences, Sari, Iran; 4 Center for Research and Training in Skin Diseases and Leprosy, Tehran University of Medical Sciences, Tehran, Iran; 5 Department of Community Medicine, Faculty of Medical Sciences, Iran University of Medical Sciences, Tehran, Iran; 6 Department of Microbiology, Faculty of Medical Sciences, Iran University of Medical Sciences, Tehran, Iran; 7 Department of Medical Parasitology and Mycology, School of Medicine, Shahid Beheshti University of Medical Sciences, Tehran, Iran

**Keywords:** *Candida albicans*, Expression, Filamentous growth, *TUP1* gene, Vulvovaginal candidiasis

## Abstract

**Background and Purpose:**

Recurrent vulvovaginal candidiasis (RVVC) is one of the most common gynecological conditions in healthy and diabetic women, as well as antibiotic users. The present study was conducted to determine the relationship between *TUP1* gene expression patterns and symptomatic recurrent *C. albicans* infections

**Materials and Methods:**

This research was performed on *C. albicans* samples isolated from the vaginal specimens obtained from 31 individuals with *RVVC* in 2016. The reference strain *C. albicans* ATCC 10231, 10 *C. albicans* strains isolated from minimally symptomatic patients, and 10 isolates from asymptomatic patients were also used as control strains. The relative mRNA expression of the *TUP1* gene was quantified using quantitative real-time polymerase chain reaction (QRT-PCR)

**Results:**

The QRT-PCR results revealed that *TUP1* mRNA expression was significantly decreased (0.001-0.930 fold) in the *C. albicans* isolates obtained from RVVC patients (*P*<0.001). However, no *TUP1* expression was detectable in the isolates collected from asymptomatic patients. The results also indicated a significant correlation between *TUP1* mRNA expression level and the severity of itching and discharge (*P*<0.001)

**Conclusion:**

The present results were suggestive of the probable contribution of *TUP1*, as a part of the transcriptional repressor, to the severity of the symptoms related to *C. albicans* infections in the vagina. Regarding this, it is required to perform more in vivo studies using a larger sample size to characterize the regulatory or stimulatory function of *TUP1* in the severity of RVVC symptoms. Furthermore, the study and identification of the genes involved in the severity of the symptomatic manifestations of *C. albicans*, especially in resistant strains, may lead to the recognition of an alternative antifungal target to enable the development of an effective agent

## Introduction

Recurrent vulvovaginal candidiasis (RVVC) is one of the most common gynecological conditions in healthy [ 1- 3] and diabetic women, as well as antibiotic users [ 4 , 5]. Currently, 5-8% of women worldwide suffer from RVVC, with a minimum recurrence rate of 4 episodes per year [ 5 , 6]. Although *Candida albicans* has been considered the primary cause of RVVC, emerging evidence increasingly points to the causal role of non-*Candida albicans Candida* (NCAC) species, particularly *C. glabrata* [ 7 , 8 ].

The frequent occurrence of RVVC in different populations without identifiable predisposing factors highlights the role of unknown possible genetic deficiencies in the host, as well as the pivotal virulence factors of the pathogenic fungi [ 3]. Several characteristics of *C. albicans* are directly involved in the pathogenesis of this species [ 9 - 13]. For instance, the transition between yeast and filamentous growth is one of the most outstanding virulence factors [ 14 , 15]. There is a hypothesis suggesting that the filamentous form is more invasive than the yeast form [ 16] because it can penetrate the tissue and escape from the immune system of the host [ 17].

It seems that this morphogenesis reflects the integrity/interaction of multiple genetic and environmental factors responsible for full virulence. The morphogenesis also emphasizes the necessity of the recognition of the genes involved in morphogenesis and accounting for pathogenesis during symptomatic recurrent infections, especially in patients with unknown underlying diseases. It is believed that hyphal formation is controlled by a panel of transcriptional activators (e.g., EFG1 and CPH1) [ 18 , 19 ] and co-repressor complexes (e.g., TUP1, NRG1, and RFG1) [ 10].

In addition, several signaling pathways, such as mitogen-activated protein kinase (MAPK or MAP kinase), Ras/cyclic AMP signaling, calcium/ calmodulin-dependent pathways, and some environmental conditions are also involved [ 20]. According to the literature, the deletion of *TUP1*, *EFG1*, and *CPH1* genes could induce constitutive filamentous growth [ 21 , 23]. Moreover, the mutation of *TUP1* reportedly contributed to the increase of the expression levels of several genes, such as *ALS* and *SAP*, promoting *C. albicans* and increasing virulence [ 24]. However, the activation of *TUP1* transcription repressor complexes results in the repression of ﬁlament-speciﬁc gene expression [ 24].

The exact mechanism of the functional role of *TUP1* in morphogenic switching is controversial. There is a bulk of evidence indicating that *TUP1* directly represses filamentation or encodes a panel of repressor genes to induce filamentous formation [ 23]. However, the exact relevant factors, which are responsible for the incidence of symptomatic and recurrent *C. albicans* infections, are not known yet.

Experimental evidence regarding hyphal formation suggests that *TUP1* is involved in morphogenesis via various signaling pathways and encodes the genes promoting mucosal pathogenesis [ 23]. Hence, a hypothesis arose from the fact that *TUP1* expression might have a stimulatory effect on symptomatic pathology. With this background in mind, the present study was conducted as the first attempt to determine the correlation between *TUP1* gene expression patterns and symptomatic recurrent vulvovaginal candidiasis caused by *C. albicans* using the QRT-PCR.

## Materials and Methods

**Study design and participants**

The present experimental study was performed on 31 *C. albicans* samples isolated from vaginal 

specimens obtained from 31 individuals with RVVC (with the presence of filament in the direct examination of vaginal discharge) in 2016 [ 3]. In addition, *C. albicans* ATCC 10231, 10 *C. albicans* strains isolated from minimally symptomatic patients, and 10 isolates from asymptomatic patients were used as control strains.  

The ability of all 31 strains to develop filaments were confirmed on the yeast extract peptone dextrose (YEPD) broth without any pretreatment (Merck, Germany) at 39°C for 1 h [ 25, 26]. The symptoms of RVVC patients, including itching and discharge, were monitored by physician visits and recorded precisely during the sample collection. In addition, the identification of the clinical control isolates was performed according to a previous study performed by Ghazanfari et al. [ 3].

**Primer design**

The PCR primers were designed using online Primer3 software (version 0.4.0) (http://primer3.ut.ee.) and synthesized by the Bioneer Company (Korea; Table1).

**Table1:** Primer sequences used in the study.

Gene name	Primer	Nucleotide sequence (5'→3')
*TUP*	*TUPF*	*TCAAGGAAATCCCACCATTC*
	*TUPR*	*AATCTCACGCAGCAAACAAC*
*ACT*	*ACTF*	*GCTGTTTTCCCATCTCTTGTT*
	*ACTR*	*GCTTCGGTCAACAAAACTGG*

**RNA Extraction**

To ensure the relationship of mRNA expression with the infection and promote hyphal growth, total RNA (for clinical isolates and ATCC strain) was extracted in the early stage of mycelia growth (log-phase) on the YEPD broth without any pretreatment (Merck, Germany) at 39°C (25, 26), using the RNX-plus solution (Cinnagen, Iran). The qualities and concentrations of the extracted RNA were checked with agarose gel electrophoresis and a spectro-photometer (ND-1000, Thermo Scientific Fisher, US), respectively. In order to remove any DNA contamination, the RNA was treated by DNase1 (Fermentas, USA) according to the manufacturer’s instructions.

**Complementary DNA Synthesis**

Complementary DNA (cDNA) was synthesized using 3 μg RNA, 20 pmoles/μL random hexamer (Fermentas, Burlington, Canada), and 20 pmoles/μL Oligo-dT (Fermentas, Burlington, Canada) [ 27]. Subsequently, it was incubated at 65°C for 5 min and then added with 10 μL Hyperscript RT Master Mix (GeneAll, Korea). In the next stage, the sample was kept at 25°C for 5 min, followed by incubation at 42°C for 60 min and finally warmed up to 85°C for 5 min.

The integrity of the cDNA was checked using the housekeeping gene ACTIN primers. The PCR denaturation process was carried out for 5 min at 96°C, 45 sec at 94°C, 45 sec at 60°C, and 1 min at 72°C for an extension of 30 cycles. Finally, it was heated up to 72°C for 10 min. In the next stage, all appropriate cDNAs were stored at -20°C.

***Quantitative real-time polymerase chain reaction***

The QRT-PCR was conducted in duplicate with 20 µl volumes using the Q-Master Mix SYBR Green I (2X) (Ampliqon, Denmark) and RG-3000 (Corbett, Australia). Subsequently, 1 μL single-stranded cDNA was added to a microtube, containing 10 μL Q-Master Mix SYBR Green I (2 X), 0.8 μL of each of the forward and reverse *TUP1* primers (10 pmoles/μL), and H_2_O of up to 20 μL. The thermal protocol was performed by activation at 95°C for 15 min, followed by amplification at 95°C for 35 sec and 55°C for 40 sec for 35 cycles. The *ACT* gene was used as a house-keeping agent to normalize the data. In order to ensure the accuracy of the examination, the average *TUP1* mRNA expression level of the control strains (i.e., reference strain and clinical strains isolated from patients with minimally symptomatic RVVC) was measured and used as the baseline.

The results were analyzed using the comparative Ct method (ΔΔCt) by REST© software (2009, version 2.0.13). Study approval was obtained from the Ethics Committee of Iran University of Medical Sciences, Tehran, Iran (NO. 26990).

**Statistical analysis**

Data analysis was conducted in SPSS software, version 16 (SPSS, Chicago, IL, USA) using Fisher’s exact and Mann-Whitney U tests. A *P-value* less than 0.5 was considered statistically significant.

## Results 

**Figure 1:**
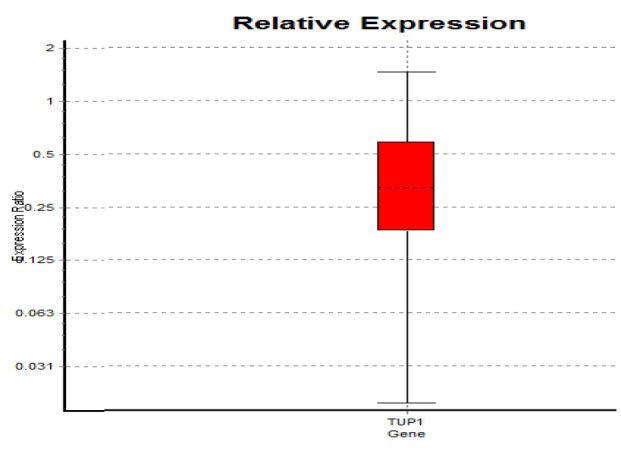
Comparison of the expression of *TUP1* at mRNA level between 31 samples and control group (*P*<0.001).

The itching and discharge were reported in 17 (54.83%) and 8 (25.80%) RVVC patients, respectively. Furthermore, 6 (19.35%) cases developed both itching and discharge simultaneously. The QRT-PCR results revealed a significant decrease (0.001-0.930 fold) in the *TUP1* mRNA expression in all 31 *C. albicans* isolates (*P*<0.001) in comparison to that in the control group (Figure 1). In addition, a significant 

correlation was observed between *TUP1* mRNA expression level and the severity of itching and discharge (*P*<0.001).

## Discussion

The results of the present study demonstrated a significant downregulation in *TUP1* mRNA expression levels in the isolates with filamentous growth in the microscopic specimen of vaginal discharge after 1 h of growth in the *YEPD* broth at 39°C, compared to that in the control specimens. This finding, along with moderate to severe clinical manifestations, suggests a direct correlation between the downregulation of *TUP1* mRNA expression with hypha formation and the severity of symptomatic recurrent vaginal infections. In other words, it seems that highly symptomatic infections are associated with a higher down regulation level of *TUP1* mRNA and vice versa.

Moreover, the importance of *TUP1* level in *C. albicans*-induced infection severity has been shown in corneal infections. The *RBT4* gene expression level and/or downstream of *TUP1* refer to the present results. There was a relationship between the higher expression level of *TUP1* mRNA and the presence of asymptomatic recurrent infections. These findings were obtained from the comparison of the results obtained from symptomatic patients with asymptomatic controls with no expression of *TUP1* mRNA and confirm our hypothesis (i.e., a relationship between the higher expression level of *TUP1* mRNA and the presence of asymptomatic recurrent infections).

Two explanatory hypotheses were raised from the recent imaging of *TUP1* gene expression. Firstly, EFG1, as a key activator of filamentation, could interfere with *TUP1* expression via the inhibition of NRG1 expression. Secondly, possible genetic changes in the inhibitory complexes (e.g., *NRG1*, *RFG1*, and *TUP1*) can inhibit *TUP1* gene expression. 

## Conclusion

The present results were suggestive of the contribution of *TUP1*, as a part of the transcriptional repressor, to the severity of symptoms related to *C. albicans* infections in the vagina. However, it is required to perform more in vivo studies on a larger sample size to characterize the regulatory or stimulatory function of *TUP1* in the severity of RVVC symptoms. Furthermore, the study and identification of the genes involved in the severity of the symptomatic manifestations of *C. albicans*, especially those of resistant strains, may lead to the recognition of an alternative antifungal target to facilitate the development of an effective agent. 
